# A retropharyngeal multinodular goiter: A case report and literature review

**DOI:** 10.1016/j.ijscr.2022.107122

**Published:** 2022-04-27

**Authors:** Alhanouf A. Alhedaithy, Abdulaziz M. AlGhamdi, Tariq H. Abualhamayel, Nada A. Aldabal

**Affiliations:** King Fahad Medical Military Complex, Dhahran, Saudi Arabia

**Keywords:** CT, computed tomography, ORL, otorhinolaryngology, OR, operating room, Multinodular goiter, Thyroid gland, Neck mass, Euthyroid, Thyroidectomy, Dyspnea

## Abstract

**Introduction:**

Thyroid goiter is a benign chronic enlargement of the thyroid gland, which presents as a painless anterior neck mass with occasional extension to the mediastinum. Retropharyngeal goiter is a rare presentation and hardly reported in the literature.

**Presentation of case:**

A 70-year-old male presented with a multinodular goiter with a large retropharyngeal component.

**Clinical findings and investigations:**

Physical examination of the head and neck revealed a massive anterior neck mass. Laryngeal endoscopy with a 70-degree rigid endoscope demonstrated a large retropharyngeal mass completely obstructing the view of the larynx. Computed tomography (CT) scan revealed a large multinodular goiter with suprahyoid and retrosternal extension, resulting in displacement of the trachea.

**Interventions and outcome:**

The mass was excised completely under general anesthesia and intubation was done under fiberoptic bronchoscopy guidance. The mass was sent for histological analysis, which confirmed the diagnosis of Hashimoto thyroiditis.

**Conclusion:**

Eventually, upon follow-up at three months post excision, no evidence of recurrence was detected.

## Highlights

• A 70-year-old male presented with a multinodular goiter with a large retropharyngeal component.
• The goiter was excised completely under general anesthesia and intubation was done under fiberoptic bronchoscopy guidance
• The goiter was sent for histological analysis, which confirmed the diagnosis of Hashimoto thyroiditis.
• Postoperative fiberoptic laryngoscopy revealed a significantly improved airway with bilateral symmetrical moving vocal cords.

## 1 Introduction

Thyroid goiter is a non-neoplastic chronic enlargement of the thyroid gland, which presents as a painless anterior neck mass with occasional extension to the mediastinum. The incidence of thyroid goiter in developed countries is approximately 4%, but this is around 10% in countries with iodine deficiency [1]. Thyroid goiter is usually managed medically, which often reduces the size of the thyroid gland and prevents disease progression. However, if a goiter is clinically unnoticed or if treatment was delayed or ineffective, this can cause the gland to enlarge beyond its confines, thus requiring surgical removal. Most often, thyroid goiters primarily extend caudally into the mediastinum. However, retropharyngeal goiter is rarely reported in the literature [2], [3], [4], [5], [6], [7]. Physical examination of the head and neck using a fiberoptic laryngoscope and via computed tomography (CT) scan can confirm the diagnosis of a retropharyngeal goiter. Herein, we present the case of a 70-year-old male with a multinodular goiter with a large retropharyngeal component.

## 2 Presentation of case

This work has been reported in line with the SCARE 2020 criteria [8].

**Fig. 1:**
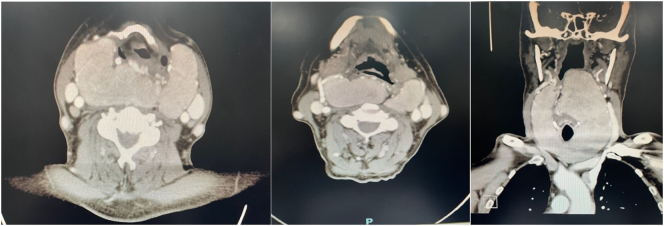
Large retropharyngeal goiter seen in imaging. CT scan of the neck revealing a large thyroid goiter. Right hyoid bone fracture is noted.

**Fig. 2:**
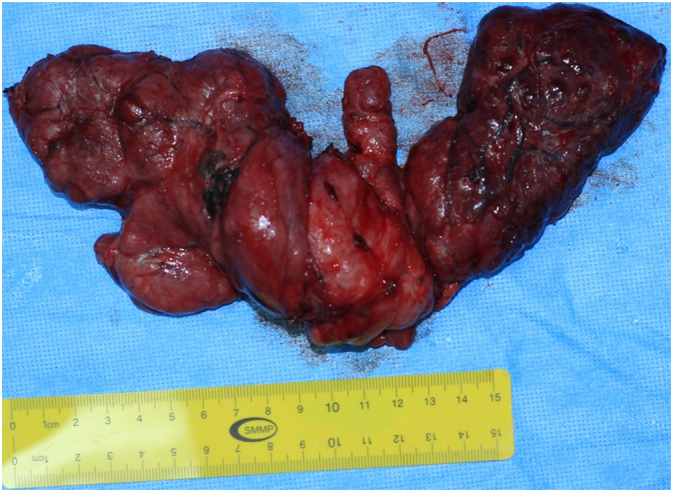
Resection of the thyroid goiter. Gross appearance of the excised thyroid gland. The gland was diffusely nodular.

A 70-year-old male presented to the otorhinolaryngology (ORL) outpatient clinic at King Fahad Medical Military Complex in Al Dhahran, Saudi Arabia, for the evaluation of an anterior neck mass. He first noticed the mass one year prior to presentation. At that time, the mass was painless but was gradually increasing in size. He denied dysphagia, changes in voice quality, dyspnea, or signs of hypo- or hyperthyroidism. He also denied weight loss, night sweats, and had no history of recent foreign travel or radiation exposure. He was otherwise healthy and reported a long history of smoking but no alcohol consumption. No family history of malignancies was reported. On physical examination, there was a large firm painless anterior neck mass which did not move with deglutition. Laryngeal endoscopy with a 70-degree rigid endoscope demonstrated a huge retropharyngeal mass indenting the posterior pharyngeal wall at the level of the oropharynx which completely obstructed the view of the larynx. Thyroid function tests revealed that the patient was euthyroid. On CT scan, there was a large multinodular goiter with suprahyoid and retrosternal extension, resulting in mass effect upon the surrounding structures including displacement of the trachea (Fig. 1). Total thyroidectomy under general anesthesia to prevent airway compromise was presented as a treatment option and discussed with the patient. The surgery and its outcomes were explained to the patient, and written informed consent was obtained. Total thyroidectomy was performed and intubation in the operating room (OR) was done under fiberoptic bronchoscopy guidance. An enormous lobulated thyroid gland extending into the retropharyngeal space was extracted with no intraoperative complications (Fig. 2). Postoperative fiberoptic laryngoscopy revealed a significantly improved airway with bilateral symmetrical moving vocal cords. Histological examination revealed features consistent with a Hashimoto thyroiditis with no evidence of malignancy. The patient's postoperative recovery was uneventful.

## 3 Discussion

The thyroid gland lies in the anterior triangle of the neck, situated within the pretracheal layer and attached to the trachea via the ligament of Berry. The pretacheal layer of fascia extends between the hyoid bone superiorly and the mediastinum inferiorly. It has two parts: the muscular part which encloses the infrahyoid muscles, and the visceral part which encloses the thyroid gland, trachea, and esophagus. The posterior aspect of the visceral fascia is formed by contributions from the buccopharyngeal fascia (fascia covering the pharynx). The retropharyngeal space is located between the buccopharyngeal fascia and the prevertebral fascia; it extends from level of the skull base to the posterior mediastinum.

The superior extension of the thyroid gland is limited by the superior attachment of the pretracheal fascia and the attachment of the infrahyoid muscles. Due to anatomic compartments created by fascial planes within the neck, goiters tend to grow into the mediastinum where it follows the path of least resistance, resulting in substernal or retrosternal goiter. This causes symptoms related to compression of the airway and/or esophagus. In a minority of patients, mediastinal extension is followed by growth superiorly behind the oro-hypopharynx, which may produce dysphagia and dysphonia [9], [10]. It is rare for a goiter to grow and extends superiorly, presenting as retropharyngeal mass, as in our case.

In conducting a literature review, seven cases of retropharyngeal goiter have been reported [2], [3], [4], [5], [6], [7]. (Table 1) Dyspnea was the most common chief complaint reported, followed by dysphagia and dysphonia. Despite the large size of the goiter in our case, our patient had asymptomatic presentation. Additionally, most of the reported cases were histologically identified as multinodular goiters. Only one patient had Hashimoto's thyroiditis, as in our case [3]. Among the previously reported cases, only was managed with thyroid suppression therapy, which resulted in symptomatic improvement [3].

**Table 1:** Clinical characteristics of previously reported cases of retropharyngeal goiter.

	Age	Sex	Symptom/s	Thyroid hormone level	Clinical size of thyroid gland	Management
Soboroff, B. J. (1977)	42	F	Noisy breathing	Euthyroid	Enlarged	Total thyroidectomy
Kenyon (1981)	57	M	Change in voice	Hypothyroid	Normal	Biopsy
Som and Sugar (1991)	79	F	Dyspnea	Euthyroid	Enlarged	Total thyroidectomy
Som and Sugar (1991)	71	F	Dyspnea	Euthyroid	Enlarged	Total thyroidectomy
Atasoy, Çetin (2002)	78	M	Dysphagia Dysphonia	Euthyroid	Enlarged	Total thyroidectomy
Lakhani (2010)	63	M	Dysphagia Dysphonia Dyspnea	Euthyroid	Enlarged	Total thyroidectomy
Carissa (2017)	53	M	Dysphagia Dyspnea	Not mentioned	Enlarged	Total thyroidectomy
Our patient (2021)	70	M	Asymptomatic	Euthyroid	Enlarged	Total thyroidectomy

In the present patient, due to the impending compressive symptoms our patient was managed surgically via a classical total thyroidectomy, and intubation in the OR was done under fiberoptic bronchoscopy guidance as there was a concern for upper airway obstruction and laryngeal compression.

## 4 Conclusion

Our case demonstrates the importance of understanding neck spaces in relation to visceral structures. In patients with thyroid goiter, it is important to recognize the possibility of a retropharyngeal or mediastinal extension of the thyroid gland and consider these as differential diagnoses of a neck mass. CT imaging of the neck is recommended in such presentations and surgical intervention is the treatment of choice to prevent airway obstruction. In addition, a multidisciplinary team approach with careful preoperative evaluation using fiberoptic laryngoscopy and an extensive discussion of the potential plans to secure the airway with the anesthesia team will prevent poor outcomes.

## Sources of funding

This research did not receive any specific grant from funding agencies in the public, commercial, or not-for-profit sectors.

## Consent

Written informed consent was obtained from the patient for publication of this case report and accompanying images. A copy of the written consent is available for review by the Editor-in-Chief of this journal on request.

## Ethical approval

The case report was approved by the institutional review board at King Fahad Medical Military Complex, Dhahran, Saudi Arabia.

## Provenance and peer review

Not commissioned, externally peer-reviewed.

## Author contributions

Alhanouf Alhedaithy: Wrote the manuscript. Part of the surgical team.

Abdulaziz AlGhamdi: Part of the surgical team.

Tariq Abualhamayel: Operated the patient, Reviewed the manuscript.

Nada A Aldabal: Operated the patient, Reviewed the manuscript.

## Registration of research studies

Not applicable.

## Guarantor

Alhanouf Alhedaithy.

## Declaration of competing interest

None.
